# Short- and long-term outcome differences between patients undergoing left and right colon cancer surgery: cohort study

**DOI:** 10.1007/s00384-024-04623-w

**Published:** 2024-05-04

**Authors:** Justas Kuliavas, Kristina Marcinkevičiūtė, Augustinas Baušys, Klaudija Bičkaitė, Rimantas Baušys, Vilius Abeciūnas, Austėja Elžbieta Degutytė, Marius Kryžauskas, Eugenijus Stratilatovas, Audrius Dulskas, Tomas Poškus, Kęstutis Strupas

**Affiliations:** 1 Clinic of Gastroenterology, Nephrourology and Surgery, Institute of Clinical Medicine, Faculty of Medicine, Vilnius University, LT-03101 Vilnius, Lithuania; 2 Department of Abdominal and General Surgery and Oncology, National Cancer Institute, 1 Santariskiu str., LT-08406 Vilnius, Lithuania; 3 Faculty of Medicine, Vilnius University, 21/27 M. K. Ciurlionio str., LT-03101 Vilnius, Lithuania

**Keywords:** Colon cancer, Right colectomy, Left colectomy, Complications, Anastomotic leak

## Abstract

**Purpose:**

Since the literature currently provides controversial data on the postoperative outcomes following right and left hemicolectomies, we carried out this study to examine the short- and long-term treatment outcomes.

**Methods:**

This study included consecutive patients who underwent right or left-sided colonic resections from year 2014 to 2018 and then they were followed up. The short-term outcomes such as postoperative morbidity and mortality according to Clavien-Dindo score, duration of hospital stay, and 90-day readmission rate were evaluated as well as long-term outcomes of overall survival and disease-free survival. Multivariable Cox regression analysis was performed of overall and progression-free survival.

**Results:**

In total, 1107 patients with colon tumors were included in the study, 525 patients with right-sided tumors (RCC) and 582 cases with tumors in the left part of the colon (LCC). RCC group patients were older (*P* < 0.001), with a higher ASA score (*P* < 0.001), and with more cardiovascular comorbidities (*P* < 0.001). No differences were observed between groups in terms of postoperative outcomes such as morbidity and mortality, except 90-day readmission which was more frequent in the RCC group. Upon histopathological analysis, the RCC group’s patients had more removed lymph nodes (29 ± 14 vs 20 ± 11, *P* = 0.001) and more locally progressed (pT3-4) tumors (85.4% versus 73.4%, *P* = 0.001). Significantly greater 5-year overall survival and disease-free survival (*P* = 0.001) were observed for patients in the LCC group, according to univariate Kaplan-Meier analysis.

**Conclusions:**

Patients with right-sided colon cancer were older and had more advanced disease. Short-term surgical outcomes were similar, but patients in the LCC group resulted in better long-term outcomes.

## Introduction

Colorectal cancer (CRC) stands as the world’s 3rd most prevalent cancer and the 4th leading cause of cancer-related deaths [1]. The conventional approach combines right-sided and left-sided colorectal cancer for evaluating treatment outcomes [2, 3]. Considering the colon’s development from the midgut and hindgut [4], it exhibits variations in embryonic origin concerning vascular and nerve supply, microbial load, and primary physiological functions of the left and right colons. Consequently, the location of the tumor may be important in influencing pathogenesis, development, and overall outcomes [5, 6].

The surgical procedures for right and left hemicolectomies differ in technique and complexity, potentially leading to different complication rates. Although right hemicolectomy was considered to be a simpler procedure because of the omission of colocolic or colorectal anastomosis and better postoperative results than left hemicolectomy [7–9], current literature presents diverse data on short- and long-term outcomes. Some studies suggest that patients after left colectomy face more postoperative complications, including increased surgical site infections, a higher incidence of ureteral injuries, and a greater conversion rate to open surgery, along with an extended hospital stay [10], especially for elderly patients due to anastomotic leak that also leads to higher mortality risk [11]. Conversely, other reports argue that complications are more frequent in patients undergoing right colectomy [12]. Results from the American College of Surgeons National Surgical Quality Improvement Program database show that mortality and major complication rates were similar between both groups [8].

Given the conflicting data in today’s literature regarding postoperative outcomes in right and left hemicolectomies, we conducted this study to compare short- and long-term treatment results.

## Methods

### Ethics

Vilnius Regional Ethics Committee approved the study (No. 2019/3-1116-608, 2019-03-20) before it was conducted. A waiver for informed consent was given with respect to the retrospective nature of the study. All study-related procedures were performed following the Declaration of Helsinki of 1975, as revised in 1983.

### Patients, diagnostic pathway, surgery, and follow-up

The study was carried out at two major colorectal cancer treatment centers in Lithuania: the National Cancer Institute and Vilnius University Hospital Santaros Klinikos. All consecutive patients who underwent surgical treatment for colon cancer from January 2014 to December 2018 were screened for inclusion in the study. Patients with multiple colon tumors and patients who underwent surgery without primary anastomosis were excluded. The study included all patients who did not meet the exclusion criteria.

The standardized diagnostic pathway for colon cancer patients involved colonoscopy and biopsy, followed by chest, abdominal, and pelvic computed tomography (CT). Once the diagnosis of colon cancer was confirmed, and staging was completed, all patients underwent thorough discussion in multidisciplinary team meetings. For patients without distant metastases, radical surgery was typically scheduled. The decision to proceed with initial surgery or surgery after neoadjuvant chemotherapy in cases of metastatic disease was personalized based on individual considerations. After surgery, all patients were allocated for medical-oncologist consultation, and chemotherapy was administered based on individual case.

The type of surgery depended on tumor location and typically, tumors located on the right colon, hepatic flexure, or middle part of the transverse colon were treated with right colectomy, while tumors on the left-side colon, splenic flexure, and sigmoid colon were resected by left colectomy or sigmoid resection [13], although, the exact extent of surgery and the approach (open or laparoscopic) were selected by a surgeon. The standard follow-up protocol consisted of a carcinoembryonic antigen (CEA) blood marker and computed tomography (CT) scan every 3 months for the first 2 years, then biannually and annually until 5 years after surgery or patient’s death. A colonoscopy was performed 1 year after surgery.

### Study outcomes

For comparison, patients were grouped into right colon cancer (RCC) and left colon cancer (LCC) groups based on tumor localization as mentioned above. The short-term study outcomes included: postoperative morbidity according to Clavien-Dindo score [14] and mortality, length of stay, and 90-day readmission rate. Long-term outcomes included overall survival (OS) and disease-free (DFS) survival. OS was defined as the time between diagnosis of colon cancer and death. DFS was defined as the time from diagnosis to the recurrence of disease.

## Statistical analysis

All statistical analyses were conducted using the statistical program SPSS 25.0 (SPSS, Chicago, IL, USA). Continuous variables are presented as the mean ± standard deviation (SD) and were compared across groups using independent samples *t*-test. Categorical variables are shown as proportions and compared using the *χ*^2^ or Fisher exact tests, as appropriate. OS and DFS rates were analyzed by the Kaplan-Meier method and were compared between the study groups by the log-rank test. Multivariable Cox proportional hazard regression analysis was used to identify the factors impacting long-term outcomes. Hazards ratios (HRs) were presented with 95% confidence intervals (CI). In all statistical analyses, two-tailed tests were used and a *P*-value of < 0.05 was considered to be significant.

## Results

### Baseline characteristics

**Fig. 1:**
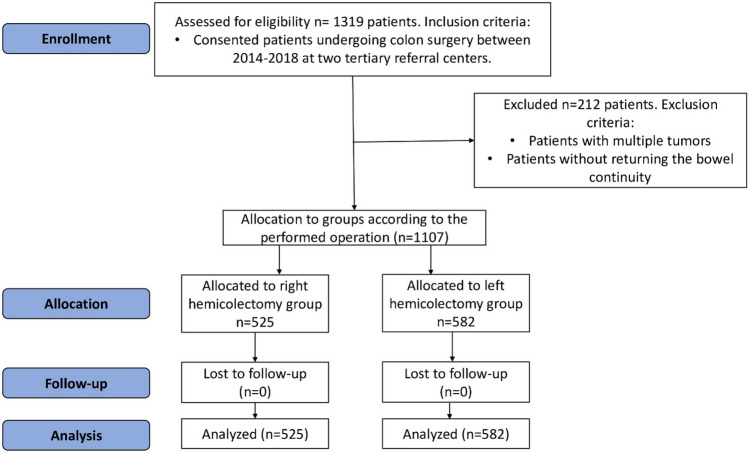
A flow chart of included and excluded patients

In total, 525 (47.4%) patients were included in the RCC group and 582 (52.6%) patients in the LCC group (Fig. 1).

The baseline characteristics of the groups are shown in Table 1. Patients in the RCC group were slightly older, had higher ASA scores and a higher proportion of these patients had cardiovascular comorbidities.

**Table 1:** Baseline characteristics of patients in RCC and LCC groups

	RCC (*n* = 525)	LCC (*n* = 582)	*P* value
Age (years), mean ± SD	69 ± 11	66 ± 10	0.001
Sex, *n* (%)			0.001
Male	219 (41.7%)	307 (52.7%)	
Female	306 (58.3%)	275 (47.3%)	
American Society of Anesthesiologists score, *n* (%)			0.001
ASA 1-2	232 (44.2%)	378 (64.9%)	
ASA 3-4	293 (55.8%)	204 (35.1%)	
Comorbidities, *n* (%)			
Cardiovascular	271 (51.6%)	154 (26.5%)	0.001
History of stroke	16 (3.0%)	18 (3.1%)	0.999
Chronic renal failure	11 (2.1%)	12 (2.1%)	0.999
Diabetes	71 (13.5%)	70 (12.0%)	0.471
Stage of disease, *n* (%)			0.001
I	66 (12.6%)	128 (22.0%)	
II	193 (36.8%)	195 (33.5%)	
III	193 (36.8%)	192 (33.0%)	
IV	73 (13.9%)	67 (11.5%)	
Tumor localization, *n* (%)			N/A
Caecum	131 (25.0%)		
Ascending colon	264 (50.3%)		
Hepatic flexure	67 (12.8%)		
Proximal transversum	63 (12.0%)		
Distal transversum and splenic flexure		57 (9.8%)	
Descending colon		71 (12.2%)	
Sigmoid		326 (56.0%)	
Regtosigmoid junction		128 (22.0%)	

*ASA* American Society of Anesthesiologists score, *N/A* not applicable

### Surgical outcomes

Surgical outcomes and histology are shown in Table 2. A higher proportion of patients in the LCC group received minimally invasive surgery. Postoperative morbidity (24.7% vs 27.1%, *P* = 0.366) and mortality (2.1% vs 1.9, *P* = 0.807) rates between RCC and LCC groups were similar. Ninety-day re-admission rate was higher in the RCC group (6.3% vs 2.1%, *P* = 0.001). Eleven patients in the right hemicolectomy group had an anastomotic leak, 1 was treated conservatively, and 10 were reoperated, while 41 patients in the left hemicolectomy group had an anastomotic leak, 11 were reoperated, 1 was drained, 1 was closed with an Ovesco staple, and 28 were treated conservatively. Histological examination showed that patients in the RCC group had a higher number of retrieved lymph nodes (29 ± 14 vs 20 ± 11, *P* = 0.001) and more locally advanced (pT3-4) tumors (85.4% vs 73.4, *P* = 0.001).

**Table 2:** Surgery and postoperative outcomes of patients in RCC and LCC groups

	RCC (*n* = 525)	LCC (*n* = 582)	*P* value
Type of surgery, *n* (%)			N/A
Right colectomy	483 (92.0%)	1 (0.2%)	
Transversum resection	26 (5.0%)	2 (0.3%)	
Left colectomy	3^a^ (0.6%)	174 (29.9%)	
Sigmoidectomy/high anterior resection	0 (0%)	401 (68.9%)	
Total colectomy	11 (2.1%)	2 (0.3%)	
Segmental resection	2 (0.4%)	2 (0.3%)	
Length of surgery (minutes), mean ± SD	144 ± 51	139 ± 55	0.173
Surgical approach, *n* (%)			0.001
Open	437 (83.2%)	299 (51.4%)	
Minimally invasive	88 (16.8%)	283 (48.6%)	
Conversion rate, *n* (%)	9 (1.7%)	16 (2.7%)	0.247
Blood loss (ml), mean ± SD	130 ± 130	89 ± 137	0.377
Length of specimen (cm), mean ± SD	33 ± 14	21 ± 11	0.001
Retrieved lymph nodes number, mean ± SD	29 ± 14	20 ± 11	0.001
Pathological tumor stage, *n* (%)			0.001
pT1/2	77 (14.6%)	155 (26.6%)	
pT3/4	448 (85.4%)	427 (73.4%)	
Pathological nodal stage, *n* (%)			0.031
pN0	272 (51.8%)	339 (58.2%)	
pN+	253 (48.2%)	243 (41.8%)	
Radicality of surgery			0.942
R0	524 (99.8%)	581 (99.8%)	
R1-2	1 (0.2%)	1 (0.2%)	
Patients with postoperative complications, *n* (%)	130 (24.7%)	158 (27.1%)	0.366
Clavien-Dindo complications score			0.877
CD1/2	77 (14.7%)	95 (16.3%)	
CD ≥ 3	53 (8.0%)	63 (8.9%)	
90-days readmission rate, *n* (%)	33 (6.3%)	12 (2.1%)	0.001
Postoperative mortality, *n* (%)	11 (2.1%)	11 (1.9%)	0.807

*N/A* not applicable, *CD* Clavien-Dindo, *pT* pathological tumor stage, *pN* pathological nodal status

^a^The tumor was in the middle of the transverse colon

### Long-term outcomes

**Fig. 2:**
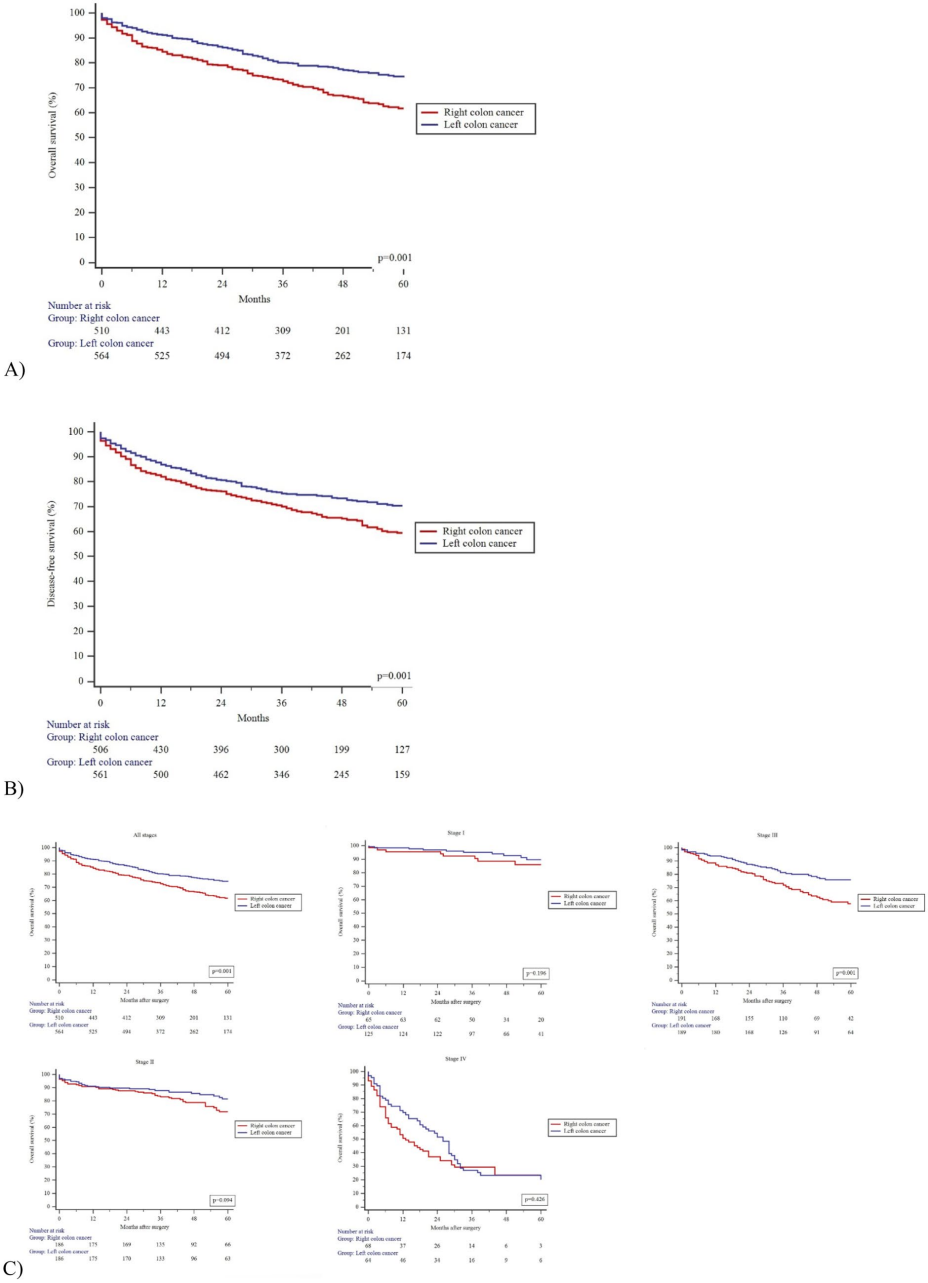

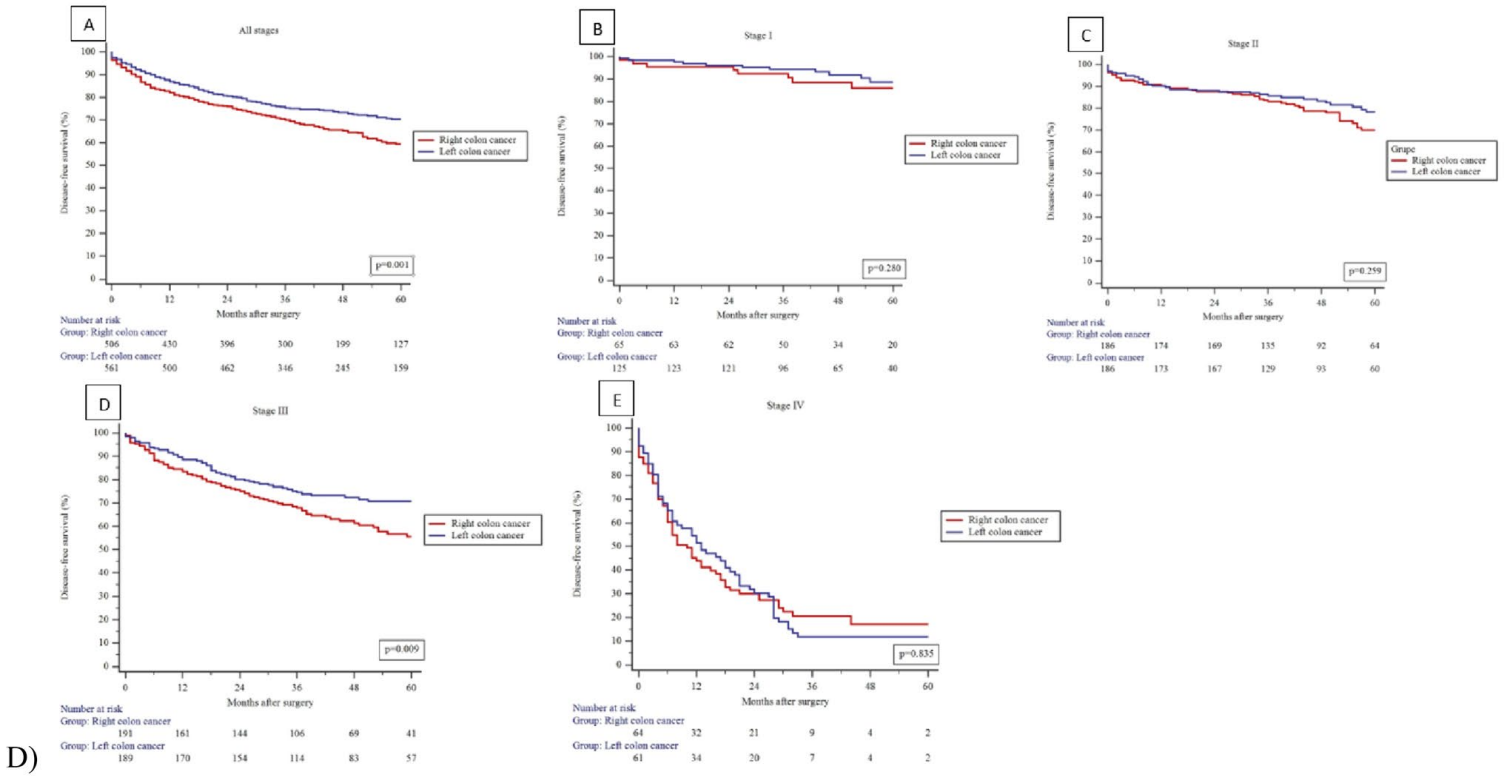
Kaplan-Meier curves. **A** Overall survival (OS). **B** Disease-free survival (DFS). **C** Subgroups according to each stage of colon cancer in OS. **D** Subgroups according to each stage of colon cancer in DFS

The mean follow-up time was 43 ± 22 months. Univariate Kaplan-Meier analysis showed a significantly higher 5-year OS (61.7% vs 74.1%; *P* = 0.001) and DFS (59.4% vs 70.4%; *P* = 0.001) for the patients in LCC group (Fig. 2). Although, multivariable Cox regression analysis demonstrated no evidence that the risk of death or disease progression was higher in RCC after adjustment for age, stage of the disease, and ASA score (Table 3).

**Table 3:** Multivariable Cox regression analysis of overall and progression-free survival

Variable	Category	Overall survival		Progression-free survival	
		HR (95% CI)	*P* value	HR (95% CI)	*P* value
Tumor localization	RCC	1 (Reference)		1 (Reference)	
	LCC	0.801 (0.639–1.004)	0.054	0.890 (0.720–1.102)	.286
Age		1.037 (1.025–1.050)	0.001	1.025 (1.014–1.036)	0.001
Stage of disease	I	1 (Reference)		1 (Reference)	
	II	1.742 (1.051–2.888)	.031	1.923 (1.178–3.141)	0.009
	III	2.840 (1.743–4.627)	.000	3.380 (2.107–5.422)	0.001
	IV	12.702 (7.748–20.825)	.000	16.203 (10.004–26.243)	0.001
ASA score	ASA1-2	1 (Reference)		1 (Reference)	
	ASA3-4	1.499 (1.171–1.920)	0.001	1.485 (1.175–1.876)	0.001

*HR* hazards ratio, *95% CI* 95%, *ASA* American Society of Anesthesiologists score

## Discussion

This study examined both short- and long-term outcomes in patients with right and left colon cancer. The findings revealed that individuals with tumors on the right side tended to be older and had more comorbidities. While there were no significant differences in short-term surgical outcomes, postoperative morbidity, and mortality between the two groups, a higher percentage of patients with right colon cancer experienced readmissions within 90 days postoperatively. Univariate survival analysis indicated a compromised survival rate among those with right colon cancer. This difference was attenuated after adjusting for patient age, disease stage, and physical status as represented by the ASA score.

A tendency toward higher age, greater comorbidities, higher ASA score (3/4) as well as more advanced tumors (T3/4), and higher rates of readmissions in our study was observed in the RCC group. Predominantly, minimally invasive surgery in LCC has not led to significantly lower morbidity rates compared to the more frequent open approach in RCC with additionally older and sicker patients. At the time of the study, laparoscopic operations were just entering full clinical practice in these centers. This suggests that the surgeons might still be gaining proficiency and experience with minimally invasive techniques, leading to comparable morbidity rates between the two approaches. It was noticed in the literature that the age of operated patients and the location of the tumor are closely linked. The older the patient is, the more proximal to the ileocecal valve the tumor is [8, 12, 13, 15–20]. Patients were predominantly older in the RCC group most likely due to a delay in the detection of right-sided colon cancer. Cancer on the right side of the colon might present with more modest symptoms than cancer on the left side, which is frequently associated with anemia and weight loss, while left-sided colon cancer tends to cause partial bowel obstruction leading to constipation, narrowed stool, diarrhea, abdominal pains, tenesmus, bloating, and visible rectal bleeding, or complete bowel obstruction needing emergency treatment [13, 21]. Moreover, in some countries younger patients undergo screening sigmoidoscopy (UK guidelines), and more tumors in the left colon side are detected earlier than symptoms start [22]. Despite that the time from cancer onset to clinical symptoms is considered to be similar for both sides of the colon — between 4.5 and 5.8 years [23], our study shows that RCC group patients present with more advanced tumors (T3-4). As a result, it can lead to more difficult surgery, worse outcomes, and more frequent re-hospitalization due to complications. Study shows that advanced cancer is a significant risk factor for anastomotic leakage, and therefore to poorer outcomes [24]. On the other hand, there is evidence in the literature that tumors on the right and left sides of the colon have completely different molecular and histological characteristics. Mutations in the DNA mismatch repair pathway are often found in RCC tumors, which are more sensitive to immunotherapy and less sensitive to chemotherapy, whereas LCC tumors are usually characterized by molecular mutations in the chromosomal instability pathway, which is better affected by chemotherapy [25]. That suggests that tumor pathogenesis and likely outcomes depend on the anatomical location. Therefore, more clinical trials are needed to confirm the underlying pathway.

Previous studies summarizing outcomes after right or left colectomy have shown mixed results. A study showed no significant differences in short- and long-term outcomes [26]. Comparable results, but only a higher readmission rate in the RCC group, similar to our trial, were noticed in the study of evaluation of laparoscopic surgery in hemicolectomies [27]. However, there are controversial data on postoperative complication rates in comparison to “traditional” resection for colon cancer, and complete mesocolic excision (CME) — that is similar to Japanese D3. The goal of this approach is to preserve the integrity of embryological planes and ensure a complete lymphadenectomy [28, 29]. To ensure proper staging of the disease, according to the Union for International Cancer Control (UICC) recommendations, a minimum of 12 lymph nodes should be removed and examined [30]. Typically, the right colon contains a significantly greater number of lymph nodes compared to the left [31]. Therefore, as fewer lymph nodes are normally in the left colon, it is commonly recommended to perform central vascular ligation (CVL) in left hemicolectomy to achieve CME and obtain the highest possible number of retrieved lymph nodes [29, 32]. Studies show that D3 right hemicolectomy also results in a larger harvested lymph node yield without increasing morbidity and even mortality [33, 34]. The need for CME in right colectomy surgery is therefore debated. Some authors claim that CME is linked to higher intraoperative organ injuries that lead to longer hospitalization and poorer short-term outcomes [35], but others report that there was no difference in overall complication rates with CME [36]. Nevertheless, recent studies and ongoing trial results have shown that extended lymphadenectomy has oncological benefits in colorectal cancer for better staging [35, 37]. Moreover, that results in decreased local recurrence rates and thus in lower overall mortality rates [29, 33].

Our study has some limitations. First, we excluded patients without primary anastomoses (for example patients with large left-sided tumors that may have had a Hartmann-type operation) as this could influence the results — taking into account the fact that most probably these patients may have been the most frail ones. Second, we did not evaluate the quality of surgery — although highly skilled colorectal surgeons with experience of at least 100 colectomies performed most of the surgeries and the complete mesocolic excision with high vascular ligation is the gold standard at both institutions. Moreover, we have not assessed the nutritional state of the patients and the molecular mutations of the cancer as well as the genetic and molecular and immunobiological factors. Additionally, we could not retract the data on adjuvant chemotherapy; this might have the effect on long-term survival. Finally, we did not have the data on the reasons for 90-day readmission.

## Conclusion

This study evaluated the short- and long-term outcomes for patients with right- and left-sided colon cancer. The results showed that people with right-sided cancer were often older and had more comorbidities, although, both groups did not significantly vary in terms of postoperative morbidity, mortality, or short-term surgical outcomes. However, higher 5-year overall survival and disease-free survival were observed for the patients in the LCC group. Thus, age, comorbidities, and tumor stage are the main determinants of a patient’s prognosis after hemicolectomy. Further research is necessary to identify other possible factors affecting outcomes after right or left colectomy.

## Data Availability

Patients’ data is stored in the authors’ database. Data is not publicly available.
